# Microencapsulated Propionate and Butyrate Improved Energy Balance and Gut Microbiota Composition in Diet-Induced Obese Rats

**DOI:** 10.3390/nu17132180

**Published:** 2025-06-30

**Authors:** Souvik Patra, Prasanth K. Chelikani

**Affiliations:** School of Veterinary Medicine, Texas Tech University, 7671 Evans Drive, Amarillo, TX 79106, USA; soupatra@ttu.edu

**Keywords:** microencapsulated short chain fatty acids, energy balance, gut microbiota, metabolome

## Abstract

Short-chain fatty acids (SCFA), particularly propionate and butyrate, reduce food intake, body weight, and modulate gut microbiota composition. However, it is unclear whether encapsulation of propionate and butyrate enhances their delivery to distal gut to improve energy balance and gut microbiota composition in obesity. **Objectives**: We determined the effects of microencapsulated propionate and butyrate on energy balance, gut microbiota and metabolite composition in obese rats. **Methods**: In experiment-1, obese male Sprague-Dawley rats were fed microencapsulated propionate and butyrate (5–10% *wt*/*wt*) or control high-fat diet. In experiment-2, obese rats were fed 10% microencapsulated propionate and butyrate, non-encapsulated butyrate (10% *wt*/*wt*), and pair-fed to non-capsulated butyrate. Food intake, energy expenditure (EE), sympathetic-mediated EE changes by propranolol, body composition, gut microbiota and fecal and plasma metabolites were analyzed. **Results**: Microencapsulated propionate decreased caloric intake, weight and fat mass, while microencapsulated butyrate had modest effects. Non-encapsulated butyrate reduced intake and prevented weight gain beyond pair-fed controls. Microencapsulated propionate and non-encapsulated butyrate reduced respiratory quotient suggestive of a shift towards lipid utilization, and enhanced β-adrenergic-mediated EE. Microencapsulated propionate and butyrate altered alpha and beta diversity metrics, microencapsulated propionate increased *Verrucomicrobiae*, microencapsulated butyrate increased *Lactobacillaceae*, and microencapsulated propionate and butyrate reduced *Erysipelotrichia*. Microencapsulated propionate and butyrate increased fecal amino acids and altered select plasma metabolites; microencapsulated propionate increased fecal and plasma propionate, supporting distal gut delivery. **Conclusions**: Dietary supplementation with microencapsulated propionate and butyrate improved energy balance, enhanced lipid utilization, beneficially modulated gut microbiota composition, and altered metabolite profiles in obese rats.

## 1. Introduction

Fermentation of fiber in the distal gut by microbes generates the primary short-chain fatty acids (SCFA)—acetate, propionate and butyrate, which, in turn, modulate host physiology and metabolism [1]. Of the SCFAs, propionate and butyrate have garnered considerable attention for their anti-obesity properties [2,3,4]. Propionate and butyrate impact host metabolism by regulating satiety and lipid oxidation, and influencing immune function [1,2]. In both rodent models and human studies, supplementation with either propionate or butyrate has been shown to prevent weight gain and improve insulin sensitivity but the effects on food intake are inconsistent. For instance, dietary butyrate administration at 5% *wt*/*wt* in mice has been reported to reduce food intake, curb weight gain, improve insulin sensitivity [5,6], and increase energy expenditure and fat oxidationy [5],whereas, a similar dose of sodium butyrate in the diet [7] or 0.1M in the drinking water [8] lowered body weight without altering food intake in mice. Sodium butyrate supplementation, together with caloric restriction, was reported to increase the feelings of fullness and reduce weight and BMI, in obese adult humans [9]. Similarly, propionate at 4% *wt*/*wt* suppressed food intake and weight gain in mice [6]. In humans, dietary sodium propionate significantly enhanced satiety in some [10,11,12] but not in all studies [13], delayed gastric emptying [14], reduced postprandial blood glucose and insulin levels [14,15], and increased energy expenditure and fat oxidation [12,16]. These beneficial effects partly stem from how butyrate and propionate each reshape the gut microbiota. For instance, butyrate supplementation in high-fat diet (HFD)-fed rodents is particularly effective at enriching *Ruminococcaceae*, *Lachnospiraceae*, *Blautia*, *Erysipelotrichaceae* and *Lactobacillus*—taxa known for their fermentative capacity and SCFA production [17,18,19], and propionate increased the relative abundance of *Bacteroidetes* and *Verrucomicrobia* while decreasing *Firmicutes* phyla [20,21]. However, whether the SCFA generated from microbial fermentation in the distal gut directly engage host neurohumoral mechanisms to improve host energy balance and metabolic health is poorly understood.

Despite encouraging evidence on the anti-obesity properties of propionate and butyrate, their efficacy remains inconsistent, owing likely to differences in dosage, delivery strategy and host-specific factors [4,22]. A potential contributing factor to these mixed results is that free SCFAs, including propionate and butyrate, are rapidly absorbed in the jejunum and ileum [23,24,25], and also in the colon with the colonocytes showing a higher preference for butyrate [26,27,28,29], thus limiting their systemic availability to exert host-specific metabolic benefits. To address this challenge, techniques were developed that either esterified SCFA with a prebiotic fiber or microencapsulated the SCFA in lipid matrices to protect SCFAs from proximal intestinal digestion and target their release to the large intestine [30]. For example, in humans, inulin–propionate ester at 10 g/day significantly reduced food intake, weight gain, body fat accumulation [31,32] and at 20 g/day improved insulin sensitivity and altered gut microbiota composition by increasing the relative abundance of *Actinobacteria* and decreasing *Clostridia* at the class level [33]. The consumption of microencapsulated butyrate was reported to reduce the severity of clinical symptoms of diverticulitis, irritable bowel syndrome [34,35,36], and inflammatory bowel disease in adult patients [37], decrease waist circumference and improve insulin sensitivity in obese children [38], and decrease blood pressure in adult diabetic patients [39]. In production animals and birds, dietary supplementation of encapsulated sodium propionate to broiler chicken suppressed food intake, abdominal fat deposition and altered cecal microbiota composition [40]. Further, encapsulated butyrate (1000 mg/kg) protected against *Clostridium perfringens*-induced intestinal damage in broiler chicken [41], improved indices of mucosal barrier function in Holstein calves [42] and increased beneficial taxa and reduced pathogen colonization in turkeys [43] and pigs [44]. While these findings collectively underscore the potential of targeted SCFA delivery to the distal gut to improve metabolic and microbial outcomes, key questions remain with regards to the optimal dose, duration and/or mode of delivery, and whether the supplementation of encapsulated propionate and butyrate improve host metabolism and gut microbial composition in animal models of human obesity.

We hypothesized that dietary microencapsulated propionate and butyrate will improve energy balance and gut microbiota composition in a diet-induced obese rat model of human obesity. Our objectives were to first determine the dose-dependent effects of microencapsulated propionate and butyrate on food intake, body weight and composition. We next assessed the acute effects of microencapsulated propionate and butyrate on energy intake and expenditure, and gut microbiota composition and fecal and plasma metabolite concentrations. Lastly, we determined whether β-adrenergic receptor signaling plays a role in the alterations in energy expenditure by microencapsulated propionate and butyrate.

## 2. Materials and Methods

### 2.1. Animal Experiments

Male Sprague Dawley rats (*n* = 28, starting weight 65 ± 4.8 g (g), 4 week (wk) old, Charles River Laboratories, Wilmington, MA, USA) were group housed under controlled laboratory conditions (i.e., 25 ± 2 °C, 40–50% humidity, 12-h (h) light-dark cycle with lights off at 1230 h). Rats had ad libitum access to tap water and food. All experimental procedures were approved by Texas Tech University Health Sciences Center Institutional Animal Care and Use Committee (IACUC protocol# 21022), and the ARRIVE reporting guidelines were followed [45]. The rats were group housed in microisolator cages and fed a control high-fat diet (HFD, 4.63 kcal/g, 40% fat kcal) [46] for 2 months to induce obesity. All diets were prepared in-house according to AIN-93 guidelines [47] and stored at 4 °C until further use. For sample size, based on data from our previous studies [48,49,50], in a randomized design with α = 0.05, the effect size, SD, power and sample size for body weight are 0.93, 15.53 g, 99%, 8 rats; and for body fat are 1.49, 4.37 g, 100%, 6 rats, as generated by Systat11^®^ (Systat Software Inc., Palo Alto, CA, USA). The authors were not blinded to the group allocation during animal experimentation and data analyses.

**Figure 1:**
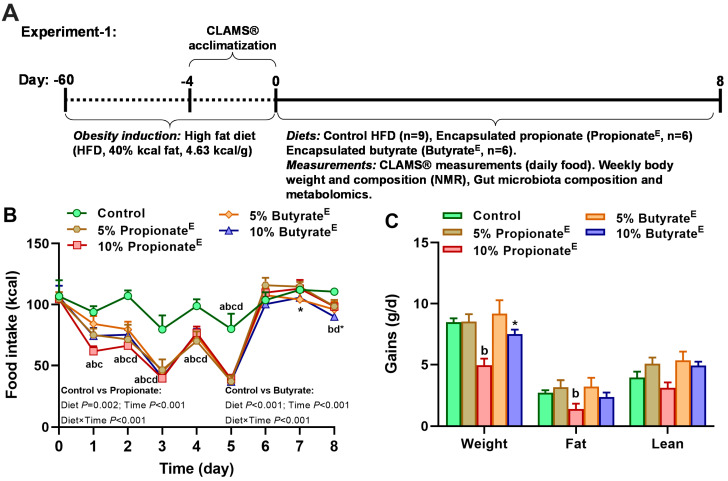
(**A**) Timeline for experiment-1. Obesity was induced in male Sprague Dawley rats with a high-fat diet (HFD, 40% fat kcal) for ~60 days in microisolator cages. Rats were next acclimatized to CLAMS^®^ cages for 4 days, and then randomized (*n* = 6–9/group) to the following dietary groups: control (*n* = 9, 40% fat kcal), propionate^E^ (5 and 10% microencapsulated propionate *wt*/*wt*, *n* = 6/group, 44 and 49% fat kcal) and butyrate^E^ (5 and 10% microencapsulated butyrate *wt*/*wt*, *n* = 6/group, 43 and 45% fat kcal) for 8 days. Measurements included food intake, respiratory quotient (RQ), energy expenditure (EE) in CLAMS^®^, weekly body weight and body composition by NMR (Minispec LF-110), gut microbiota composition and fecal metabolite concentrations. Data from experiment-1 show (**B**) daily changes in cumulative caloric intake, (**C**) gains in weight, fat and lean mass. ^a^ *p* < 0.05; Control vs. 5% propionate^E^; ^b^ *p* < 0.05; control vs. 10% propionate^E^; ^c^ *p* < 0.05; control vs. 5% butyrate^E^; ^d^ *p* < 0.05; Control vs. 10% butyrate^E^; * *p* < 0.1; control vs. 5% propionate^E^ or 5–10% butyrate^E^; *n* = 9–6/group.

In experiment-1 (Figure 1A), we determined the dose-dependent effects of microencapsulated propionate and butyrate on food intake, body weight and fat mass. Rats (399 ± 10 g, 22% body fat; Supplemental Figure S1E,F) were acclimated and housed individually in Comprehensive Laboratory Animal Monitoring System (CLAMS^®^, Columbus Instruments, Columbus, OH, USA) for 8 days, and then assigned to the following isocaloric diets (4.63 kcal/g, Table 1) so that the average body weight was similar across groups: (1) control (*n* = 9, 40% fat kcal), (2) 5% propionate^E^ (5% microencapsulated propionate *wt*/*wt*, *n* = 6/group, 44% fat kcal; propionic acid 35% coated, SILA 398, SILA Advanced Nutrition, 30033 Noale (VE), Italy), (3) 10% propionate^E^ (10% microencapsulated propionate *wt*/*wt*, *n* = 6/group, 49% fat kcal; propionic acid 35% coated, SILA 398, SILA Advanced Nutrition, Italy), (4) 5% butyrate^E^ (5% microencapsulated butyrate *wt*/*wt*, n = 6/group, 43% fat kcal; butyrose^®^ 40% coated, SILA F-05, SILA Advanced Nutrition, Italy) and (5) 10% butyrate^E^ (10% microencapsulated butyrate *wt*/*wt*, *n* = 6/group, 45% fat kcal; butyrose^®^ 40% coated, SILA F-05, SILA Advanced Nutrition, Italy). The 10% dose was chosen for further studies due to its significant effects on food intake and body composition.

**Figure 2:**
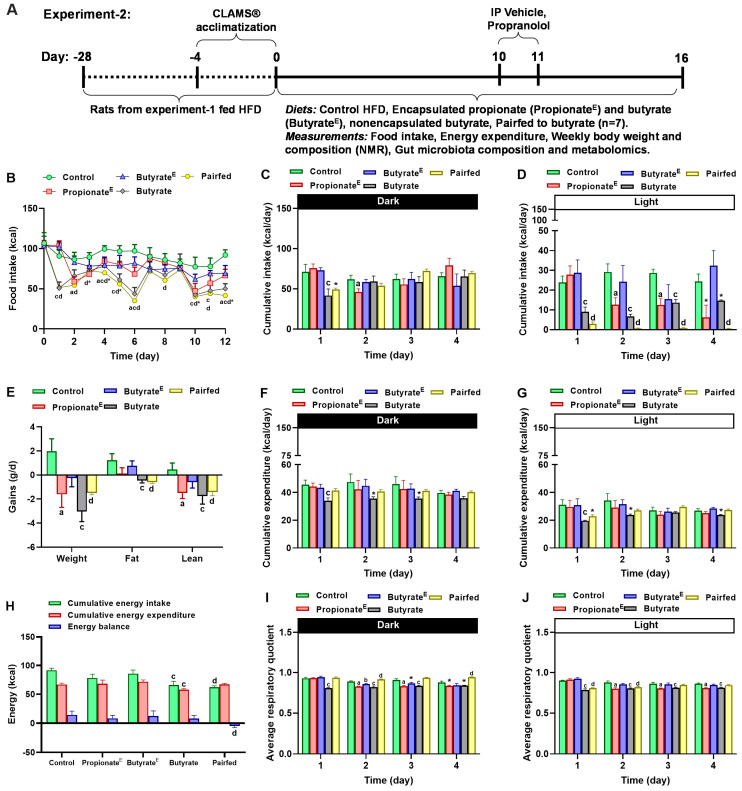
(**A**) Timeline for experiment-2. After 4 weeks on HFD adaptation, the same cohort of rats from experiment-1 were re-randomized (609.9 ± 36.7 g) to four isocaloric diets (4.63 kcal/g) for 16 days: control (*n* = 7, 40% fat), propionate^E^ (*n* = 7, 10% *wt*/*wt* microencapsulated propionate, 49% fat), butyrate^E^ (*n* = 7, 10% *wt*/*wt* microencapsulated butyrate, 45% fat), butyrate (*n* = 7, 10% *wt*/*wt* non-encapsulated sodium butyrate, 45% fat) and pair-fed group to butyrate (*n* = 7). On days 10–11, rats received intraperitoneal (IP) injections of either vehicle (0.1% BSA in 0.9% saline) or propranolol HCL (10 mg/kg BW in vehicle) in a within-subject design. On day 13, rats were fasted for ~14 h and re-fed on day 14. Measurements included food intake, respiratory quotient (RQ), energy expenditure (EE) in CLAMS^®^, and weekly body weight and body composition by NMR (Minispec LF-110), and fecal and plasma sampling on days 14 and 16, respectively. Data from experiment-2 show (**B**) daily changes in cumulative caloric intake, cumulative caloric intake for the first 4 days during the (**C**) dark phase and (**D**) light phase, (**E**) gains in weight, fat and lean mass, (**F**) changes in cumulative EE for the first 4 days during dark phase and (**G**) light phase, (**H**) average energy balance, average RQ for the first 4 days during (**I**) dark and (**J**) light phase. Values are mean ± SEM, *p* < 0.05. ^a^ *p* < 0.05; Control vs. 10% Propionate^E^; ^b^ *p* < 0.05; Control vs. 10% Butyrate^E^; ^c^ *p* < 0.05; Control vs. 10% Butyrate; ^d^ *p* < 0.05; Control vs. Pairfed; * *p* < 0.1; Control vs. 10% Propionate^E^ or Butyrate^E^ or Butyrate; *n* = 5–7/group.

In experiment-2 (Figure 2A), we determined the acute effects of 10% microencapsulated propionate and butyrate on energy balance, body weight and composition, gut microbiota composition and fecal and plasma metabolites. After 4 weeks on HFD adaptation, the same cohort of rats from experiment-1 after treatment, were re-randomized (609.9 ± 36.7, 28% body fat; Supplemental Figure S2M,N) to four isocaloric diets (4.63 kcal/g) for 16 days: (1) control (*n* = 7, 40% fat), (2) propionate^E^ (*n* = 7, 10% *wt*/*wt* microencapsulated propionate, 49% fat), (3) butyrate^E^ (*n* = 7, 10% *wt*/*wt* microencapsulated butyrate, 45% fat), (4) sodium butyrate (*n* = 7, 10% *wt*/*wt* non-encapsulated sodium butyrate, 45% fat; Fisher Scientific, Dallas, TX, USA) and (5) pair-fed to non-encapsulated sodium butyrate (n = 7). The non-encapsulated sodium butyrate group served as an internal control for the microencapsulated butyrate. The pair-fed group was provided access to the control diet in amounts identical to those consumed by the non-encapsulated sodium butyrate group. Pair-feeding was used to delineate the effects of sodium butyrate on energy expenditure independent of food intake. To investigate the role of β-adrenergic receptor signaling on energy expenditure, on days 10-11, rats received intraperitoneal (IP) injections of either (a) vehicle (0.1% BSA in 0.9% saline), or (b) propranolol HCL (10 mg/kg BW, 0.1% BSA in 0.9% saline; Sigma Aldrich, Saint Louis, MO, USA) in a within-subject design, to minimize confounds of drug effects. To assess the effects of propionate and butyrate on diet-induced thermogenesis, on day 13, rats were fasted for ~14 h and re-fed on day 14.

### 2.2. Food Intake, Energy Expenditure, Body Weight and Composition Measurements

Food consumption, energy expenditure (EE) and respiratory quotient (RQ) were monitored for 22 h (from 1230 h the previous day to 1030 h the next day) in CLAMS^®^ indirect calorimetry metabolic cages as per previously reported procedure [50,51]. These measurements were recorded from days 0–4 in experiments 1 and 2, and on days 10–14 in experiment-2. Food spillage was recorded manually and corrected for daily food intake before statistical analysis. The volume of oxygen consumed (VO_2_, ml/kg body weight/h) and carbon dioxide produced (VCO_2_, ml/kg body weight/h) were measured using an integrated Zirconia O_2_ sensor and single-beam infrared CO_2_ sensor, respectively, at frequent intervals (CLAMS setup: 2L/min flow, sampling for 1 min at every 47-min interval). The respiratory quotient was calculated as the VCO_2_:VO_2_ ratio. Total EE was computed with the following equation: Calorific value = VO_2_ × [3.815 + (1.232 × RER)] and data were reported as kcal/h. Body weight was recorded weekly using a conventional weigh scale (CL series, Ohaus Corp., Parsippany, NJ, USA). Body composition (fat and lean mass) was measured weekly with a non-invasive Minispec LF-110 NMR body composition analyzer (Bruker Corp., Billerica, MA, USA) following our previous reports [48,52,53].

### 2.3. Fecal and Cecal Microbiota Extraction, 16S Sequencing and Bioinformatics

Microbial DNA was extracted using QIAamp^®^ Fast DNA stool kit following the manufacturers instructions (Qiagen, cat# 51604, Germantown, MD, USA), from fecal samples collected on day 8 in experiment-1, and from fecal and cecal contents on days 14 and 16 in experiment-2. Briefly, ~200 mg of fecal/cecal content was collected in 2 mL sterile polypropylene microvials (cat#522s, Biospec Products, Inc., Bartlesville, OK, USA) with addition of 1 mL inhibitEx buffer and 1 mm diameter glass beads (cat#11079110, Biospec Products, Bartlesville, OK, USA), followed by homogenization using bead beater (Mini- Beadbeater-16, cat# 607, Biospec Products, Bartlesville, OK, USA) for 3 min. The extract was then incubated at 95 °C for 5 min in a thermomixer (T1317, Eppendorf Thermomixer Compact, Enfield, CT, USA) and centrifuged at 14,000 rpm for 1 min. The supernatant was then mixed with proteinase K and buffer AL and incubated at 70 °C for 10 min. 100% ethanol was then added to the mixture to precipitate the DNA. Later, samples were filtered and washed with wash buffers (AW1 and AW2) to remove impurities, and DNA was eluted with buffer ATE. The DNA samples were prepared for targeted sequencing with the Quick-16S™ Plus NGS Library Prep Kit (primer set V3–V4, Supplemental Figure S4; Zymo Research, Irvine, CA, USA). The final library was sequenced on Illumina^®^ NextSeq 2000™ with a p1 (cat# 20075294) reagent kit (600 cycles). The sequencing was performed with 30% PhiX spike-in. Unique amplicon sequences were inferred from raw reads using the Dada2 pipeline [54]. Chimeric sequences were also removed with the Dada2 pipeline. Taxonomy assignment was performed using Uclust from Qiime v.1.9.1. Taxonomy was assigned with the Zymo Research Database, a 16S database that is internally designed and curated, as a reference.

### 2.4. Metabolomics Assay

Fecal, cecal and plasma metabolites were measured by TMIC Prime assay (The Metabolomics Innovation Centre, University of Alberta, Edmonton, AB, Canada) using a combination of direct injection (DI) mass spectrometry and reverse-phase LC-MS/MS custom assay. This custom assay, in combination with an ABSciex 4000 QTrap (Applied Biosystems/MDS Sciex, Marlborough, MA, USA) mass spectrometer, was used for the targeted identification and quantification of up to 150 different endogenous metabolites including amino acids, acylcarnitines, biogenic amines and derivatives, uremic toxins, glycerophospholipids, sphingolipids and sugars, following previously reported protocol [55]. The method combines the derivatization and extraction of analytes, and the selective mass-spectrometric detection using multiple reaction monitoring pairs. Isotope-labeled internal standards and other internal standards were used for metabolite quantification. The custom assay contains a 96 deep-well plate with a filter plate attached with sealing tape, and reagents and solvents used to prepare the plate assay. For all metabolites except organic acids, samples were thawed on ice and were vortexed and centrifuged at 13,000× *g*. A total of 10 μL of each sample was loaded onto the center of the filter on the upper 96-well plate and dried in a stream of nitrogen. Subsequently, phenyl-isothiocyanate was added for derivatization. After incubation, the filter spots were dried again using an evaporator. Extraction of the metabolites was then achieved by adding 300 μL of extraction solvent. The extracts were obtained by centrifugation into the lower 96-deep well plate, followed by a dilution step with MS running solvent. For organic acid analysis, 150 μL of ice-cold methanol and 10 μL of isotope-labeled internal standard mixture was added to 50 μL of sample for overnight protein precipitation. Then it was centrifuged at 13,000× *g* for 20 min, 50 μL of supernatant was loaded into the center of wells of a 96-deep well plate, followed by the addition of 3-nitrophenylhydrazine reagent. After incubation for 2h, BHT stabilizer and water were added before LC-MS injection. Mass spectrometric analysis was performed on an ABSciex 4000 Qtrap^®^ tandem mass spectrometry instrument (Applied Biosystems/MDS Analytical Technologies, Foster City, CA, USA) equipped with an Agilent 1260 series UHPLC system (Agilent Technologies, Palo Alto, CA, USA). The samples were delivered to the mass spectrometer by an LC method followed by a direct injection method as previously described [55]. Data analysis was done using Analyst 1.6.2.

### 2.5. Statistical Analysis

Repeated measures on food intake, EE and RQ were analysed by linear mixed models using SPSS (IBM SPSS^®^ version 27, Chicago, IL, USA). The model contained fixed effects of diet, time, drug, diet × time, drug × time and drug × diet × time interaction, as appropriate. Animal within diet was considered as a random variable on which repeated measures were taken and covariance structure was selected based on the smallest Akaike’s Information Criterion (AIC) and Bayesian Information Criterion (BIC) values. Body weight and composition were analysed similarly but using baseline measures as a covariate. EE was analysed using baseline lean mass +20% fat mass as a covariate [56]. The Grubbs’ test was used to identify and exclude outliers for the various energy balance and metabolic readouts (GraphPad Prism 10.1, San Diego, CA, USA). If the main effects and/or interactions were significant, then the treatment effects were separated using Benjamini–Hochberg post-hoc analysis with a false discovery rate of 0.05 [57] (GraphPad Prism 10.1, San Diego, CA, USA). The gut microbiota composition visualization, alpha-diversity and beta-diversity analyses were performed with Qiime v.1.9.1 [58], and the Kruskal–Wallis test post hoc. Taxonomy that has significant abundance among different groups (minimum LDA score of 3) were identified by linear discriminant analysis effect size (LEfSe) [59], and PCoA plots were generated using MicrobiomeAnalyst [60]. All data were expressed as mean ± SEM, whereas, body weight and composition were represented as estimated mean from ANCOVA. Differences were considered significant at *p* ≤ 0.05, and trends at *p* ≤ 0.10.

## 3. Results

### 3.1. Microencapsulated Propionate and Butyrate Dose-Dependently Reduced Food Intake and Energy Expenditure, Shifted Substrate Utilization Toward Lipids and Decreased Body Weight Gains

In experiment-1, compared to the control (high-fat diet) group, 5 and 10% propionate^E^ decreased daily food intake by an average of 32% and 37%, respectively, between days 1 and 5. Both 5 and 10% butyrate^E^ decreased intake by 33 and 35%, respectively, between days 2 and 5, and on day 8, 10% propionate^E^ and butyrate^E^ decreased intake by 11–19%, respectively (Figure 1B). To parse out the daily intake effects, we assessed the hourly changes in cumulative intakes. Compared to the control, 5 and 10% propionate^E^ and butyrate^E^ reduced cumulative hourly caloric consumption on days 1–4 (Figure S1A–D). In support of effects on intake, we found that, compared to the control, 10% propionate^E^ reduced gains in weight and fat mass by 42–49%, without altering lean gain. The 10% butyrate^E^ tended (*p* = 0.06) to decrease weight gain by 12% without altering fat and lean gains (Figure 1C); absolute weight, fat and lean mass did not differ among groups (Supplemental Figure S1E–G). Thus, encapsulated propionate and butyrate dose-dependently decreased food intake and weight gain, and propionate at high doses also decreased adipose gain.

We next focused on the effects of high-dose propionate and butyrate on energy balance in experiment-2. As the baseline (pretreatment) food intake (Figure 2B) and body weight (609.9 ± 36.7 g) did not differ among groups, and given the protracted four-week interval between experiments, it is unlikely that there was any carryover of interventions from experiment-1 to experiment-2 on energy balance readouts. Compared to the control, 10% propionate^E^ decreased daily intake by 25–39%, 10% butyrate^E^ tended (*p* = 0.08) to decrease intake by 8–25%, 10% non-encapsulated butyrate decreased daily intake by 21–45% and as expected the pair-fed group decreased intake by 21–47% between days 1–12 (Figure 2B). To gain a better understanding of the patterns in daily caloric intakes, we assessed the temporal hourly changes in cumulative intakes. Hourly food intake showed that 10% propionate^E^ decreased intake by an average of 31% in both dark and light periods on day 2 and tended (*p* = 0.07) to decrease by 24% on day 3, 10% butyrate^E^ briefly decreased intake by 45% during 5–6 h after dark onset on day 4 and non-encapsulated butyrate and pair-fed decreased intake by 46–180% between days 1–4 compared to the control (Figure 2C,D, Supplemental Figure S2A–D).

Cumulative daily EE decreased with non-encapsulated butyrate during the dark and light periods of day 1, it tended (*p* = 0.08) to decrease on days 2-4 and tended (*p* = 0.06) to decrease in the pair-fed during the light period on day 1; propionate^E^ and butyrate^E^ did not alter cumulative EE across 4 days (Figure 2F,G). A closer examination of the timing of changes in EE within a day revealed that, consistent with daily cumulative EE changes, non-encapsulated butyrate decreased EE during the dark hours on days 1–2 with a tendency to decrease EE on days 3–4, pair-fed decreased EE during the dark hours on days 1–2 and 4, and propionate^E^ transiently decreased hourly expenditure during the dark period on days 1–2 and 4, whereas, butyrate^E^ did not alter EE (Supplemental Figure S2E–H). Compared to the control, non-encapsulated butyrate decreased overall cumulative intake and expenditure, whereas pair-fed reduced cumulative intake and energy balance with no significant change in cumulative expenditure (Figure 2H). With regards to RQ, compared to the control, propionate^E^ decreased RQ during the dark and light period on days 2–4, butyrate^E^ decreased RQ during the early dark period on day 2 and tended (*p* = 0.06) to decrease on day 3 and non-encapsulated butyrate decreased RQ during both dark and light periods across all 4 days, whereas, pair-fed increased RQ during the dark phase on day 2 and 4 and light phase on days 1–2 (Figure 2I,J; Supplemental Figure S2I–L).

Obese rats fed the control high-fat diet gained +1.98 g/d body weight, +1.2 g/d body fat and +0.46 g/d lean mass. In contrast to the control group, propionate^E^, non-encapsulated butyrate and pair-fed reduced weight gain by −1.61 g/d (or 181%), −3.1 g/d (or 254%) and −1.49 g/d (or 175%), non-encapsulated butyrate and pair-fed reduced fat mass gain by −0.46 g/d (or 138%) and −0.60 g/d (or 149%), and propionate^E^, non-encapsulated butyrate and pair-fed reduced lean gain by −1.5 g/d (or 425%), −1.75 g/d (or 478%) and −1.4 g/d (or 406%), respectively, but butyrate^E^ was ineffective (Figure 2E). Compared to the control, propionate^E^ and butyrate^E^ tended to decrease absolute body weight on day 16, whereas, propionate^E^ decreased absolute fat mass on days 8 and 16 (Supplemental Figure S2M–O).

### 3.2. Microencapsulated Propionate and Non-Encapsulated Butyrate Increased Lipid Utilization, Decreased Fasting Energy Expenditure, Yet, Sustained Sympathetically Mediated Fed Energy Expenditure

**Figure 3:**
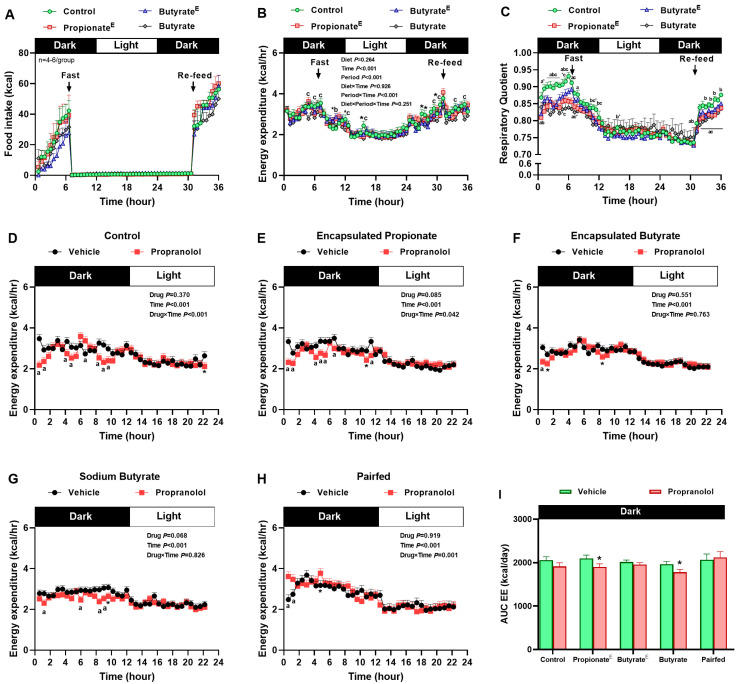
The effects of dietary microencapsulated propionate and butyrate (10% *wt*/*wt*), non-encapsulated sodium butyrate (10% *wt*/*wt*), and pair-fed to non-encapsulated butyrate on changes in hourly (**A**) food intake, (**B**) energy expenditure (EE), and (**C**) respiratory quotient (RQ) under conditions of fasting and realimentation on their respective diets. Effect of propranolol on hourly EE in obese rats fed (**D**) control, (**E**) encapsulated propionate, (**F**) encapsulated butyrate, (**G**) non-encapsulated butyrate, (**H**) pair-fed diets and (**I**) area under the curve (AUC) for EE. Values are mean ± SEM, *p* < 0.05. For panels (**A**–**C**): ^a^ *p* < 0.05, control vs. 10% propionate^E^; ^b^ *p* < 0.05, control vs. 10% butyrate^E^; ^c^ *p* < 0.05, control vs. 10% butyrate; * *p* < 0.1; control vs. 10% propionate^E^ or butyrate^E^ or butyrate; n = 5–7/group. For panels (**D**–**I**): ^a^ *p* < 0.05, vehicle vs. propanolol; * *p* < 0.1; vehicle vs. propanolol; n = 5/group.

We next assessed whether acute perturbations of energy balance by fasting and refeeding, and whether systemic β-adrenergic blockade, unmasks the latent effects of propionate and butyrate on energy expenditure and substrate utilization. When rats were fasted for 24 h and thereafter re-fed their respective diets, food intake did not differ between the groups (Figure 3A). As expected, EE decreased in all groups on fasting;, 10% propionate^E^ and butyrate^E^ tended to decrease EE during fasting, and non-encapsulated butyrate decreased EE at multiple time points during both fasting and refed state, compared to the control (Figure 3B). Notably, compared to the control, 10% propionate^E^ decreased RQ during the fed-state and tended to decrease RQ in the early fasting state, and 10% butyrate^E^ and non-encapsulated butyrate decreased RQ in both fed-state and early fasting period (Figure 3C). The β1-2-adrenergic receptor antagonist-propranolol reduced energy expenditure at multiple intervals only during the dark period compared to vehicle in control, propionate^E^, butyrate^E^ and non-encapsulated butyrate groups (Figure 3D–H). Interestingly, AUC analysis of the expenditure during the entire dark phase, revealed that propranolol decreased EE in propionate^E^ and non-encapsulated butyrate groups, but not in the control group (Figure 3I), which is supportive of enhanced sympathetic output to sustain EE in fed states.

### 3.3. Microencapsulated Propionate and Butyrate Altered Gut Microbiota Composition and Metabolite Concentrations

**Figure 4:**
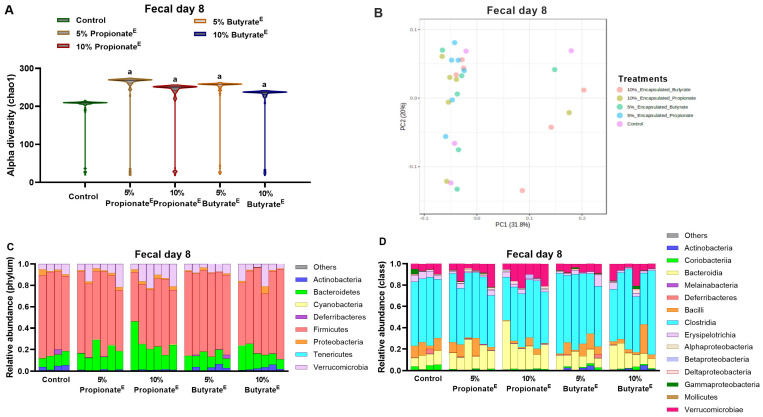
Gut bacterial compositional changes following dietary supplementation with microencapsulated propionate, microencapsulated butyrate, and non-encapsulated butyrate in obese rats. Bacterial analysis of fecal samples collected on day 8 of experiment-1 showing (**A**) alpha diversity (chao1) and (**B**) beta diversity (Bray–Curtis), and relative abundance of the gut microbiome at the (**C**) phylum level and (**D**) class level in obese rats receiving high fat control diet or microencapsulated propionate (5 or 10% propionate^E^ *wt*/*wt*) and microencapsulated butyrate (5 or 10% butyrate^E^ *wt*/*wt*). ^a^ *p* < 0.05 vs. control; *n* = 4–6/group.

In experiment-1, compared to the control, both 5 and 10% doses of encapsulated propionate and non-encapsulated butyrate significantly increased alpha diversity (chao1) in the fecal gut microbiota on day 8 (Figure 4A). The principal coordinates analysis (PCoA) plot based on Bray–Curtis dissimilarity showed that in the feces, first principal coordinate (PC1) explained 31.8% of the variance, while the second principal coordinate (PC2) accounted for 20% of the variance, with propionate^E^ and butyrate^E^ showing slight separation from the control (Figure 4B). LEfSe analysis revealed that at family level, *Rhodospirillaceae*, *Verrucomicrobiaceae* and *Rikenellaceae* in 5 and 10% propionate^E^, and *Defluviitaleaceae* in 5 and 10% butyrate^E^, were enriched to a greater extent than in the control group (Supplemental Figure S3A). Moreover, compared to the control, 10% propionate^E^ increased the relative abundance of *Bacteroidetes* and *Verrucomicrobiae* and decreased the relative abundance of *Firmicutes* at the phylum level (Figure 4C). At class level, 5% propionate^E^ decreased the relative abundance of *Erysipelotrichia* and increased *Betaproteobacteria*, 10% propionate^E^ increased *Bacteroidia*, *Betaproteobacteria*, and *Verrucomicrobiae*, but decreased *Bacilli*, *Clostridia*, *Erysipelotrichia* and 10% butyrate^E^ decreased *Erysipelotrichia* and increased *Betaproteobacteria* in the feces (Figure 4D). Fecal metabolomics analyses showed that 5% butyrate^E^ increased serine, threonine, asparagine, methionine, arginine, lysine, phenylalanine, tyrosine, tryptophan and branched chain amino acids (BCAA) valine, leucine and isoleucine. Compared to the control, 5 or 10% propionate^E^ tended (*p* = 0.06–0.07) to increase the amino acids glycine, threonine, isoleucine and arginine (Figure 6A), and 10% propionate^E^ tended (*p* = 0.06) to increase propionic acid concentrations (Figure 6B).

**Figure 5:**
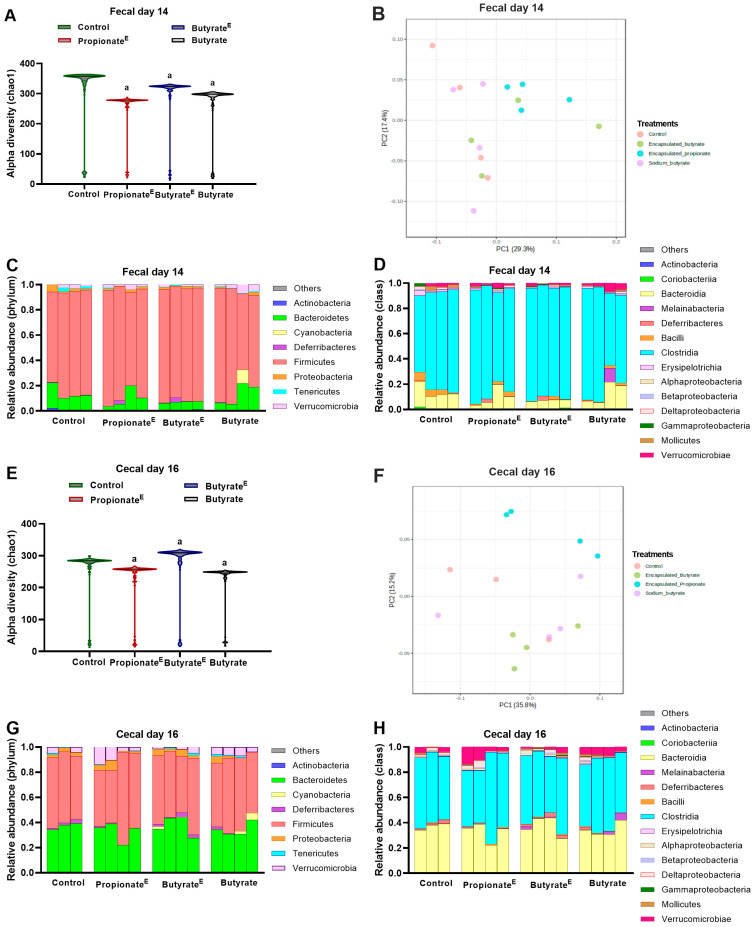
Gut bacterial compositional changes following dietary supplementation with microencapsulated propionate and butyrate, and non-encapsulated butyrate in obese rats. Fecal samples collected on day 14 from experiment-2 showing (**A**) alpha diversity (Chao1), (**B**) beta diversity (Bray–Curtis) and relative abundance of the gut microbiome at the (**C**) phylum, (**D**) class level in obese rats fed high-fat control diet, or microencapsulated propionate (10% propionate^E^ *wt*/*wt*), microencapsulated butyrate (10% butyrate^E^ *wt*/*wt*) and non-encapsulated butyrate (10% sodium butyrate *wt*/*wt*). Cecal content collected on day 16 of experiment-2 showing (**E**) alpha diversity (chao1), (**F**) beta diversity (Bray–Curtis) and relative abundance of the gut microbiome at the (**G**) phylum and (**H**) class level in obese rats fed high-fat control diet, or microencapsulated propionate (10% propionate^E^ *wt*/*wt*), microencapsulated butyrate (10% butyrate^E^ *wt*/*wt*) and non-encapsulated butyrate (10% sodium butyrate *wt*/*wt*). ^a^ *p* < 0.05 vs. control; *n* = 4–6/group.

**Figure 6:**
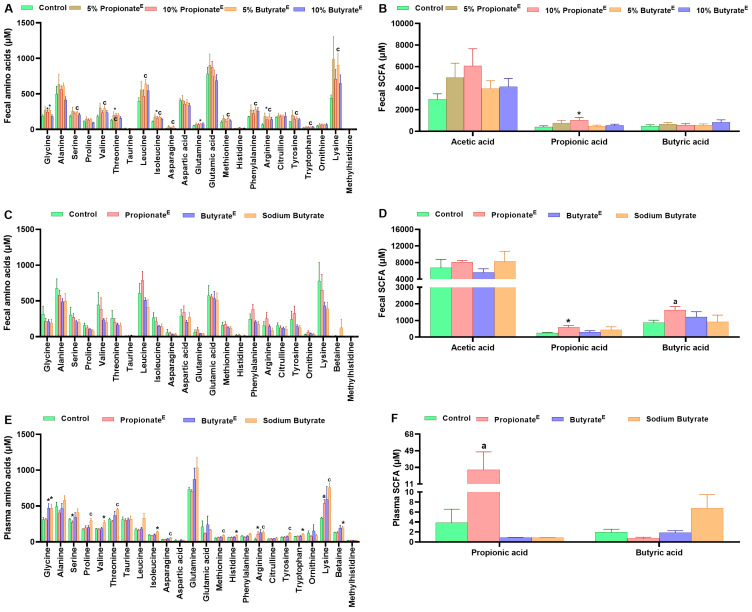
Metabolomic analysis of fecal samples collected on day 8 of experiment-1 showing concentrations of (**A**) amino acids and (**B**) short chain fatty acids (SCFA) in obese rats receiving high-fat control diet, or microencapsulated propionate (5 or 10% propionate^E^ *wt*/*wt*) and butyrate (5 or 10% butyrate^E^ *wt*/*wt*). Fecal samples collected on day 14 of experiment-2 showing concentrations of (**C**) amino acids and (**D**) SCFA. Plasma samples collected on day 16 showing concentrations of (**E**) amino acids and (**F**) SCFA in obese rats fed high-fat control diet, or microencapsulated propionate (10% propionate^E^ *wt*/*wt*), microencapsulated butyrate (10% butyrate^E^ *wt*/*wt*), and non-encapsulated butyrate (10% sodium butyrate *wt*/*wt*). Values are mean ± SEM, *p* < 0.05. ^a^ *p* < 0.05, control vs. 10% propionate^E^; ^c^ *p* < 0.05, control vs. 5% butyrate^E^ or sodium butyrate; * *p* < 0.1; control vs. 5–10% propionate^E^ or 10% butyrate^E^ or sodium butyrate; *n* = 4–6/group.

In experiment-2, compared to the control, 10% propionate^E^, butyrate^E^ and non-encapsulated butyrate decreased fecal alpha diversity (chao1) on day 14 (Figure 5A), and propionate^E^ and non-encapsulated butyrate decreased cecal alpha diversity, whereas, butyrate^E^ increased alpha diversity on day 16 (Figure 5E). PCoA plot based on Bray–Curtis dissimilarity illustrated that in feces, PC1 accounted for 29.3% of the variance, PC2 for 17.4% of the variance (Figure 5B), and in cecal contents PC1 explained 35.8% of the variance and PC2 for 15.2% of the variance (Figure 5F), with some degree of separation of treatments from control. LEfSe analysis of fecal and cecal content revealed enrichment of *Christensenellaceae* in 10% propionate^E^ and *Clostridiaceae* in 10% butyrate^E^ than in the control group (Supplemental Figure S3B,C). Compared to the control, butyrate^E^ tended to decrease the relative abundance of *Bacteroidetes* and increase *Firmicutes* at the phylum level (Figure 5C), and tended to decrease *Bacilli* and *Bacteroidia*, and increase *Clostridia* at the class level (Figure 5D). In cecal content, propionate^E^ increased the relative abundance of *Gammaproteobacteria* at the class level, but no effects were observed at the phylum level (Figure 5G,H). Regarding fecal metabolite concentration, none of the treatment groups altered amino acid concentrations (Figure 6C), but 10% propionate^E^ tended (*p* = 0.053) to increase propionic acid and increased butyric acid concentration (Figure 6D). With regards to plasma metabolites, compared to the control, propionate^E^ increased lysine and tended to increase arginine, butyrate^E^ tended to increase glycine, and butyrate increased proline, threonine, asparagine, methionine, arginine, lysine, tyrosine and tended to increase glycine, betaine, histidine, tryptophan, valine and isoleucine (Figure 6E). Notably, propionate^E^ increased plasma propionic acid concentrations compared to the control (Figure 6F).

## 4. Discussion

Dietary short-chain fatty acids (SCFA) such as propionate and butyrate, decrease food intake and body weight in animal models [18,20], however, as SCFA are primarily absorbed in the proximal gut [24,25], the effects of their distal gut delivery on energy balance are largely unknown. We compared the effects of non-encapsulated sodium butyrate, and microencapsulated butyrate and propionate which are expected to slowly release these SCFA in the lower gastrointestinal tract [61], on energy balance in obese rats. First, microencapsulated propionate and butyrate (propionate^E^, butyrate^E^), and non-encapsulated butyrate, decreased food intake, and propionate^E^ and non-encapsulated butyrate reduced body weight and adiposity gains. Second, microencapsulated propionate^E^, microencapsulated butyrate^E^ and non-encapsulated butyrate shifted substrate utilization towards fat under *ad libitum*, fasting and re-feeding states. Interestingly, microencapsulated propionate^E^ and non-encapsulated butyrate decreased fasting energy expenditure, but engaged β-adrenergic signaling to promote energy expenditure under *ad libitum* feeding conditions. Third, microencapsulated propionate^E^ and microencapsulated butyrate^E^ supplementation modulated the gut microbiota by selectively enriching beneficial fermentative taxa, and microencapsulated propionate^E^ increased fecal and plasma concentrations of propionate and select essential amino acids. Taken together, these data support the view that dietary supplementation of microencapsulated propionate and butyrate improved energy balance and gut microbiota composition in obese male rats.

Previous studies showed that the dietary or systemic administration of SCFA decreased weight and/or adiposity in rodent models and humans, but the effects on food consumption are inconsistent. For example, oral or dietary non-encapsulated propionate and butyrate (4–5% *wt*/*wt*) reduced food intake, weight and/or adiposity gains in mice [6,62], whereas, others showed that the reduction in weight and/or fat mass gain occurs without alterations in food intake [62], or that non-encapsulated butyrate increased food intake in mice [5]. Here, we demonstrated that microencapsulated propionate^E^ and butyrate^E^ dose-dependently decreased caloric intake in obese rats. A high dose of microencapsulted propionate^E^ (10% *wt*/*wt*) reduced caloric consumption and prevented weight and fat mass gain. Interestingly, microencapsulated butyrate^E^ did not alter body composition, but non-encapsulated butyrate suppressed food intake and prevented weight and fat mass gain that were greater in magnitude than in pair-fed animals. These findings indicate that the protection against adipose gain by microencapsulated propionate^E^ may be partly due to hypophagia, whereas, non-encapsulated butyrate-induced reduction in weight gain is independent of caloric intake and likely due to effects on energy expenditure or shifts in substrate utilization.

The supplementation of SCFA is purported to improve weight control by increasing energy expenditure and shifting substrate utilization towards lipids in rodents and humans [4,63], however, the findings are inconsistent among studies. For example, dietary non-encapsulated sodium butyrate at 5% *wt*/*wt* dose in mice either increased EE during the dark period [5,64] or did not alter EE [65]. However, energy expenditure in these rodent studies was either normalized to body weight [64,65] or lean mass [5], and both approaches have serious limitations [66]. In humans, oral [16] or rectal [67] administration of non-encapsulated propionate increased resting EE, decreased RQ [16] and increased fat oxidation [67], while supplementation of inulin-propionate ester tended to suppress appetite without altering resting EE and RQ [12]. In contrast to thermogenic effects of non-encapsulated sodium butyrate in mice [5,64,65], but consistent with others [65], we found that dietary microencapsulated propionate^E^ and butyrate^E^ did not alter cumulative EE during the dark and light phases, while non-encapsulated butyrate decreased cumulative EE during the dark phase in obese rats. Further, during both fasting and fed states, the reduction in EE was more pronounced in the 10% microencapsulated propionate^E^ and butyrate^E^ groups, with non-encapsulated butyrate also reducing EE. Interestingly, as the reduction in dark phase EE by propranolol was greater with microencapsulated propionate^E^ and non-encapsulated butyrate, this is supportive of a sympathetically mediated component of EE. Importantly, microencapsulated propionate^E^ and butyrate^E^, and non-encapsulated butyrate reduced RQ, indicative of a shift in substrate utilization towards lipids, and consistent with other rodent and human studies [5,16,64,65,67]. Taken together, these data suggest that microencapsulated propionate and non-encapsulated butyrate have divergent effects on energy expenditure depending on the energy balance: they induce a hypometabolic state under conditions of severe negative energy balance, but under *ad libitum* conditions microencapsulated propionate and non-encapsulated butyrate induce hypophagia but sustain energy expenditure, partly through β-adrenergic signaling.

A central finding across the two experiments is that microencapsulated propionate^E^ and butyrate^E^ reshaped the gut microbiota in distinct ways. We observed an increase in fecal alpha diversity (Chao1) within the first week, supporting the notion that microencapsulated propionate^E^ and butyrate^E^ supplementation can help maintain or enrich a more diverse microbial community [1]. However, during the second week, fecal and cecal alpha diversity declined with microencapsulated propionate^E^ and butyrate^E^, and this is in line with similar reductions in this diversity metric with other prebiotic interventions [68]. Moreover, microencapsulated propionate^E^ and butyrate^E^, and non-encapsulated butyrate, consistently modulated the beta-diversity of the gut microbiota as indicated by modest separation from the control group in PCoA plots in both experiments, suggesting that microencapsulated SCFA can reshape fermentative taxa. Strikingly, microencapsulated propionate^E^ resulted in the selective enrichment of *Verrucomicrobiaceae* and *Rikenellaceae*. Given that *Verrucomicrobiaceae* phyla include *Akkermansia muciniphila* bacteria that are known for their beneficial effects on the gut barrier function and metabolic regulation [69], this is supportive of a beneficial effect of microencapsulated propionate^E^ on the enteric microbiome. Further, the increased relative abundance of *Bacteroidetes* and decreased *Firmicutes* in the feces with microencapsulated propionate^E^ highlights the potential for specific SCFA to shift the *Firmicutes:Bacteroidetes* ratio—an indicator often linked with obesity [70]. Interestingly, microencapsulated butyrate^E^ resulted in the greater enrichment of *Lactobacillaceae* in LEfSe analyses, suggesting that butyrate release in the lower gut could shape niche environments favoring beneficial lactic acid bacteria. Microencapsulated propionate^E^ and butyrate^E^ also decreased the relative abundance of *Erysipelotrichia*, which has been associated with inflammation [71]. In addition to shifts in the microbial population, microencapsulated propionate^E^ and butyrate^E^ altered microbial protein metabolism as reflected by increased fecal amino acid excretion. This may indicate limited protein digestion and amino acid absorption, reduced bacterial deamination of amino acids, enhanced microbial utilization, and/or faster transit times that limit extensive microbial degradation [72]. Further, microencapsulated propionate^E^ tended to increase fecal propionic acid and increased plasma propionic acid concentration, which is supportive of enhanced delivery of propionate to the distal gut. As propionate increases satiety [2,33], the hypophagic effects of microencapsulated propionate^E^ in our study are consistent with the satiety from elevated circulating concentrations. In contrast to propionate, microencapsulated butyrate^E^ and non-encapsulated butyrate did not consistently increase fecal or plasma butyric acid in our studies, likely due to extensive utilization by colonocytes [73]. Previous studies reported that the consumption of microencapsulated sodium butyrate reduced signs of clinical severity of symptomatic uncomplicated diverticular disease, irritable bowel syndrome or inflammatory bowel disease [34,35,36,37,74], improved insulin sensitivity and reduced blood pressure [38,39] in human patients. Further, feeding of microencapsulated propionate or butyrate has been shown to improve the health of production animals including chicken [40,41], calves [42], turkeys [43] and pigs [44]. However, we are unaware of any reports that rigorously validated the intestinal site-specific targeted release of the SCFA from the microencapsulated products in vivo. Nonetheless, as non-encapsulated free propionate and butyrate are rapidly absorbed in the proximal gut [23,24,25], the consistent increase in plasma and fecal concentrations of propionic acid in both experiments supports enhanced release of propionate from the microencapsulated product in the distal gut. In contrast, microencapsulated butyrate altered multiple parameters of energy balance and gut microbiota composition in both experiments, which is supportive of the release of butyrate in the gut, but the doses may have been suboptimal to detect increases in plasma or fecal concentrations.

The potential limitations of the study are that the diets were not matched for carbohydrate or lipid content, and the altered ratios of carbohydrate to lipid might affect the study outcomes. However, one of the aims of our study was to formulate isocaloric diets to avoid confounds of variation in diet caloric density on food intake and energy expenditure, and assess changes in energy intake and expenditure in response to varied SCFAs. With the current diet formulation, we were able to achieve isocaloric levels across diets (i.e., 4.6 kcal/g), but a limitation with this approach was that some of the ingredient concentrations are lower than others. Though the starch content might affect the microbiome, our goal was not to assess the effects of starch concentrations *per se* on the microbiome. Notably, the total carbohydrate calories are within reasonable limits (~36–45% kcal), similar to other reports [75,76], and none of the diet groups are deficient in any one macronutrient to critically affect the microbiome. However, a potential contribution of the lower carbohydrate contents in these diets to the reduced RQ cannot be excluded. As the calorific values of propionate and butyrate were factored into the caloric contribution from fat (~40–45% kcal) across all diets, and as all diets were isocaloric, it is unlikely that the reduction in lard content contributed to the reduced weight gain of the butyrate groups. Another potential caveat of our study, as with other previous reports [34,35,36,37,38,39,40,41,42,43,44,74], is that we did not quantify the concentrations of SCFA in each segment of the intestinal tract to confirm whether the microencapsulation technique results in targeted release of SCFA in different segments of the distal gut. Although the increase in cecal and plasma SCFA concentrations and altered gut microbiota in our study are supportive of distal gut release of propionate and butyrate from the microencapsulated products, there is a need for a rigorous validation of whether the microencapsulation technique results in a controlled release of butyrate or propionate at the target site, and whether such release is impacted by other dietary and host factors.

## 5. Conclusions

Taken together, our results provide strong evidence that microencapsulated propionate and butyrate modulate energy metabolism and gut microbial communities in obese rats. While microencapsulated propionate and non-encapsulated butyrate reduced caloric intake and prevented gains in adiposity, both microencapsulated propionate and butyrate shifted substrate utilization toward fat oxidation, and microencapsulated propionate in particular engaged β-adrenergic mechanisms to drive energy expenditure. In parallel, both microencapsulated propionate and butyrate enriched potentially beneficial fermentative taxa, altered fecal amino acid profiles, and microencapsulated propionate elevated fecal and plasma propionic acid concentrations. Collectively, these findings highlight the potential of enhanced lower gut delivery of propionate and butyrate to improve energy balance with implications for the development of anti-obesity therapeutics.

## Figures and Tables

**Table 1:** Composition of diets used for experiments 1 and 2.

	Control	Propionate^E^ (5%)	Propionate^E^ (10%)	Butyrate^E^ (5%)	Butyrate^E^ (10%)	Sodium Butyrate
**Ingredients** (**g kg^−1^**)						
Corn starch ^a^	220	168	116	189	158	158
Fructose ^b^	150	150	150	150	150	150
Sucrose ^c^	150	150	150	150	150	150
Casein High Nitrogen (80-mesh) ^b^	176	176	176	176	176	176
Corn oil ^d^	60	60	60	60	60	60
Lard ^e^	145	147	149	126	107	107
α-Cellulose ^b^	50	50	50	50	50	50
Biotin ^b^	0.002	0.002	0.002	0.002	0.002	0.002
L-Cystine ^b^	1.8	1.8	1.8	1.8	1.8	1.8
Choline bitartrate ^b^	2.5	2.5	2.5	2.5	2.5	2.5
AIN-93-MX ^b^	35	35	35	35	35	35
AIN-93-VX ^b^	10	10	10	10	10	10
Sodium Propionate ^f^	0	50	100	0	0	0
Sodium Butyrate ^fg^	0	0	0	50	100	100
tert-Butylhydroquinone (tBHQ) in oil ^b^	0.008	0.008	0.008	0.008	0.008	0.008
Vanilla Extract ^h^	0.01	0.01	0.01	0.01	0.01	0.01
Total amount (g)	1000.32	1000.32	1000.32	1000.32	1000.32	1000.32
**Composition**						
Carbohydrate (% kcal) *	45%	40%	36%	42%	40%	40%
Protein (% kcal) *	15.21%	15.21%	15.21%	15.21%	15.21%	15.21%
Fat (% kcal) *	40%	44%	49%	43%	45%	45%
Energy density (kcal/g) *	4.629	4.629	4.629	4.629	4.629	4.629
Total Fiber (% wt)	5%	5%	5%	5%	5%	5%
Fat (% g/kg)	20%	26%	31%	24%	27%	27%
Mineral:Calories ratio	0.0076	0.0076	0.0076	0.0076	0.0076	0.0076
Vitamin:Calories ratio	0.0022	0.0022	0.0022	0.0022	0.0022	0.0022

^a^ PureDent B700, Ingredi, Baltimore, USA. ^b^ Dyets, Inc., Bethlehem, PA, USA. ^c^ Sucrose, Walmart Inc., Bentonville, AR, USA. ^d^ Corn oil, Walmart Inc., Bentonville, AR, USA. ^e^ Lard, Smithfield Packaged Meats Corp, Smithfield, VA, USA. ^f^ Encapsulated propionate (SILA 398, propionic acid 35% coated) and encapsulated butyrate (SILA F-05, butyrose^®^ 40% coated microencapsulated butyrate, food grade), SILA Advanced Nutrition, Italy. ^g^ Sodium butyrate, Fisher Scientific, Dallas, TX, USA. ^h^ Vanilla extract, Walmart Inc., Bentonville, AR, USA. * Energy density was calculated using calorific values (kcal/g) of 4 for carbohydrate, 4 for protein, 9 for fat, 3.8 for sodium propionate and 5.9 for sodium butyrate, respectively.

## Data Availability

The data that support the findings of this study are available from the corresponding author P. K. Chelikani upon reasonable request.

## Supplementary Materials

The following supporting information can be downloaded at: https://www.mdpi.com/article/10.3390/nu17132180/s1.

## Abbreviations

The following abbreviations are used in this manuscript: AIC—Akaike Information Criterion, AUC—Area Under the Curve, ANCOVA—Analysis of Covariance, BCAA—Branched-Chain Amino Acids, BIC—Bayesian Information Criterion, CLAMS—Comprehensive Laboratory Animal Monitoring System, EE—Energy Expenditure, GPR—G-protein-coupled receptor, HFD—High-Fat Diet, IACUC—Institutional Animal Care and Use Committee, IP—Intraperitoneal, LDA—Linear Discriminant Analysis, LEfSe—Linear Discriminant Analysis Effect Size, MX—Mineral Mix, NMR—Nuclear Magnetic Resonance, PCoA—Principal Coordinate Analysis, PX—Phylum-Level Taxonomic Classification, qPCR—Quantitative Polymerase Chain Reaction, RQ—Respiratory Quotient, SCFA—Short-Chain Fatty Acids, SEM—Standard Error of the Mean, SPSS—Statistical Package for the Social Sciences, tBHQ—Tert-Butylhydroquinone, TMIC—The Metabolomics Innovation Centre, VO_2_—Volume of Oxygen Consumed, VCO_2_—Volume of Carbon Dioxide Produced.
