# Supplementary material for: Microencapsulated Propionate and Butyrate Improved Energy Balance and Gut Microbiota Composition in Diet-Induced Obese Rats
Source: Nutrients. 2025 Jun 30;17(13):2180. doi: 10.3390/nu17132180 (PMC12251328; doi:10.3390/nu17132180)
Supplement: Supplementary file 1 [file nutrients-17-02180-s001.zip › nutrients-3664735-supplementary.pdf]

SUPPLEMENTARY INFORMATION

Title: Microencapsulated Propionate and Butyrate Improved Energy Balance and Gut Microbiota Composition in Diet-induced Obese Rats

Authors: Souvik Patra[1] and Prasanth K. Chelikani[1*]

Affiliation: [1]School of Veterinary Medicine, Texas Tech University, 7671 Evans Drive, Amarillo, Texas 79106, USA.

Keywords: Microencapsulated short-chain fatty acids, Energy balance, Gut microbiota, Metabolome

Running title: Microencapsulated short-chain fatty acids improve energy balance**.**

***Corresponding Author:** Prasanth K. Chelikani, BVSc, MVSc, PhD, FTOS, School of Veterinary Medicine, Texas Tech University, 7671 Evans Drive, Amarillo, TX 79106, USA, E-mail: pchelika@ttu.edu, phone: +1 (806) 834-5697.

Funding: This work was supported by the American Heart Association (Grant# 953881), Diabetes Research and Action Education Foundation (Grant# 523), and the Texas Tech University School of Veterinary Medicine to Prasanth K. Chelikani.

Disclosure: The authors declare no conflicts of interest.

Supplemental Figure Legends

Figure S1. The effects of dietary microencapsulated propionate (5 and 10% wt/wt) and encapsulated butyrate (5 and 10% wt/wt) on changes in hourly food intake on **(A)** day 1, **(B)** day 2, **(C)** day 3, **(D)** day 4 and on absolute **(E)** body weight, **(F)** fat mass and **(G)** lean mass. Values are mean ± SEM, $P<0.05$.

Figure S2. The effects of dietary microencapsulated propionate and butyrate (10% wt/wt), non-encapsulated sodium butyrate (10% wt/wt) and pair-fed to non-encapsulated sodium butyrate on changes in hourly food intake on representative days **(A)** 1, **(B)** 2, **(C)** 3, **(D)** 4. The effects on hourly energy expenditure on representative days **(E)** 1, **(F)** 2, **(G)** 3, **(H)** 4. The effects on hourly respiratory quotient on representative days **(I)** 1, **(J)** 2, **(K)** 3, **(L)** 4 and on absolute **(M)** body weight, **(N)** fat mass, **(O)** lean mass. Effects of intraperitoneal injection (IP) of propranolol on area under the curve (AUC) energy expenditure during the first 3 and 6 hour of dark phase in **(P)** control, **(Q)** microencapsulated propionate, **(R)** microencapsulated butyrate, **(S)** non-encapsulated sodium butyrate and **(T)** pair-fed group. Effects of propranolol on AUC EE during the **(U)** dark and **(V)** light phase in all groups. Values are mean ± SEM, $P<0.05$.

Figure S3. Gut bacterial compositional changes following dietary supplementation with microencapsulated propionate and butyrate, and non-encapsulated butyrate in obese rats. Linear discriminant analysis effect size (LEfSe) analysis showing the most abundant taxa in **(A)** fecal samples from day 8 of experiment-1 where obese rats were fed high fat control diet, 5 or 10% wt/wt of either microencapsulated propionate or butyrate. **(B)** fecal samples from day 14 of experiment-2 where obese rats were fed high fat control diet, or 10% wt/wt of either microencapsulated propionate and butyrate or non-encapsulated sodium butyrate and, **(C)** cecal samples from day 16 of experiment-2 where obese rats were fed high fat control diet, or 10% wt/wt of either microencapsulated propionate and butyrate or non-encapsulated sodium butyrate. LEfSe analysis showing the most abundant taxa in Values are mean ± SEM, $P<0.05$.

Figure S4. V3-V3 primer sequences.

Figure S1.

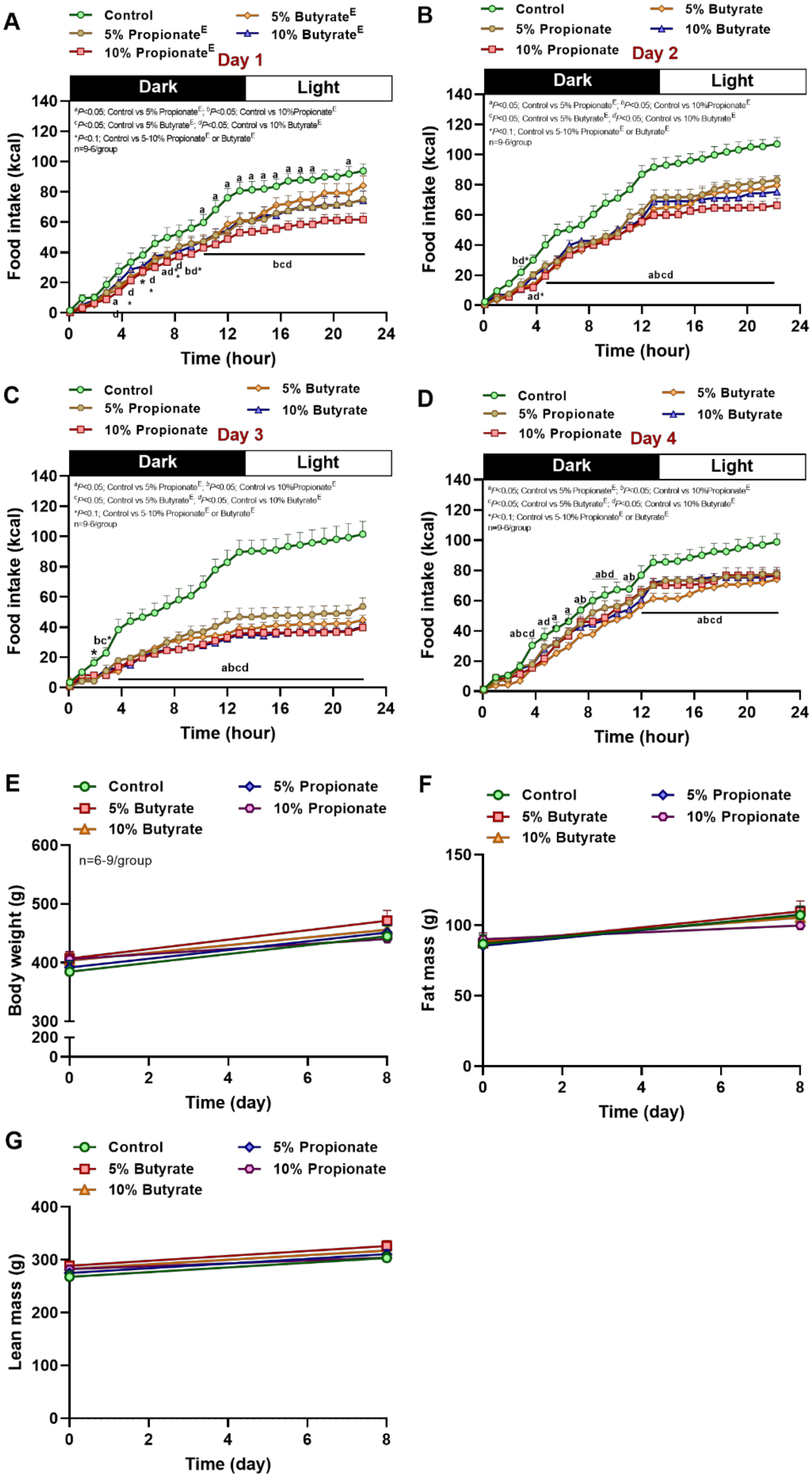
A
Control
5% Propionate^E
10% Propionate^E
5% Butyrate^E
10% Butyrate^E
Day 1
Dark
Light
^aP<0.05; Control vs 5% Propionate^E; ^bP<0.05; Control vs 10%Propionate^E
^cP<0.05; Control vs 5% Butyrate^E; ^dP<0.05; Control vs 10% Butyrate^E
*P<0.1; Control vs 5-10% Propionate^E or Butyrate^E
n=9-6/group
bcd
Food intake (kcal)
Time (hour)
B
Control
5% Propionate
10% Propionate
5% Butyrate
10% Butyrate
Day 2
Dark
Light
abcd
Food intake (kcal)
Time (hour)
C
Control
5% Propionate
10% Propionate
5% Butyrate
10% Butyrate
Day 3
Dark
Light
abcd
Food intake (kcal)
Time (hour)
D
Control
5% Propionate
10% Propionate
5% Butyrate
10% Butyrate
Day 4
Dark
Light
abcd
Food intake (kcal)
Time (hour)
E
Control
5% Butyrate
10% Butyrate
5% Propionate
10% Propionate
n=6-9/group
Body weight (g)
Time (day)
F
Control
5% Butyrate
10% Butyrate
5% Propionate
10% Propionate
Fat mass (g)
Time (day)
G
Control
5% Butyrate
10% Butyrate
5% Propionate
10% Propionate
Lean mass (g)
Time (day)

Supplementary Information: *Patra et al. Microencapsulated Propionate and Butyrate Improved Energy Balance and Gut Microbiota Composition in Diet-induced Obese Rats.*

Figure S2.

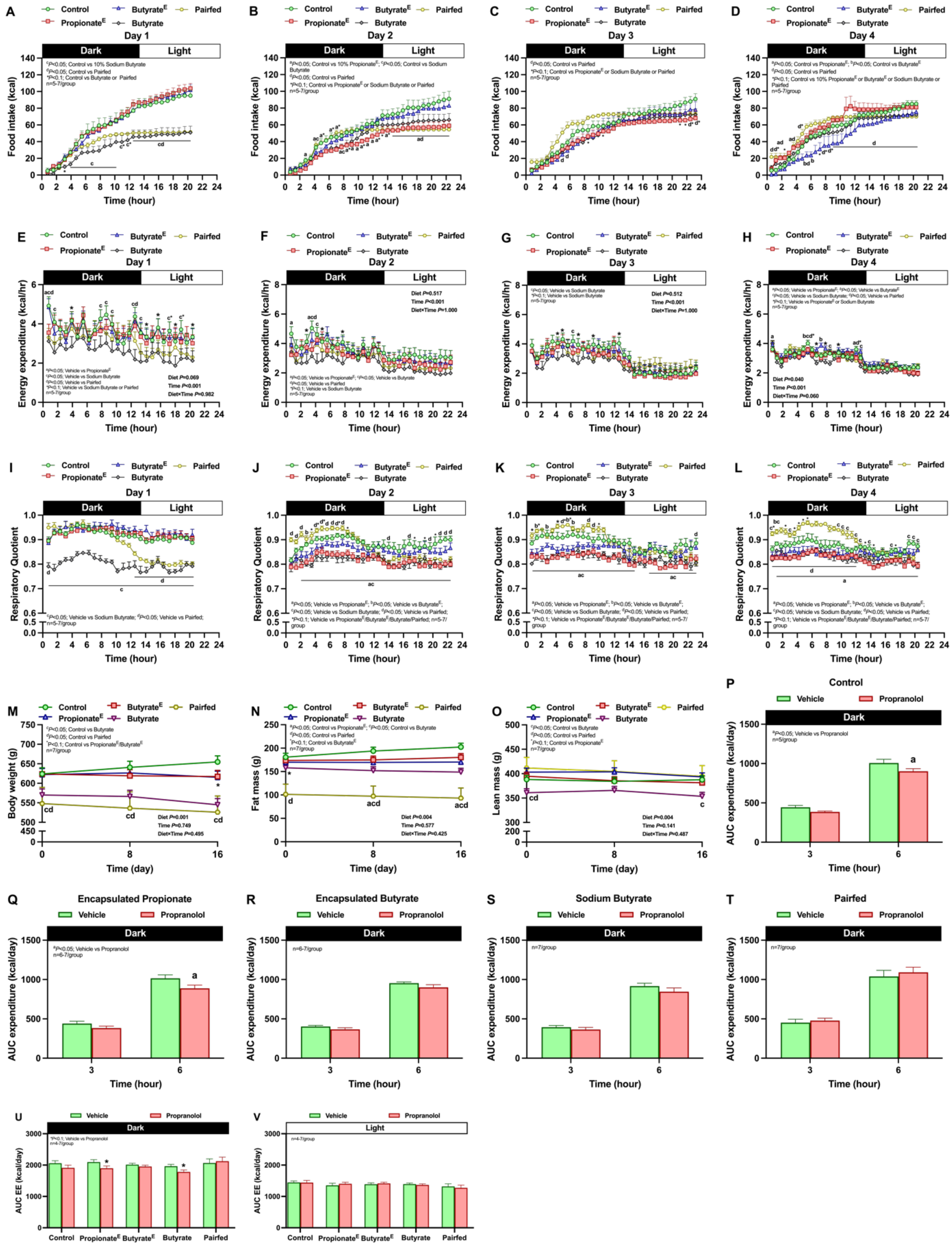

***Supplementary Information**: Patra et al. Microencapsulated Propionate and Butyrate Improved Energy Balance and Gut Microbiota Composition in Diet-induced Obese Rats.*

Figure S3.

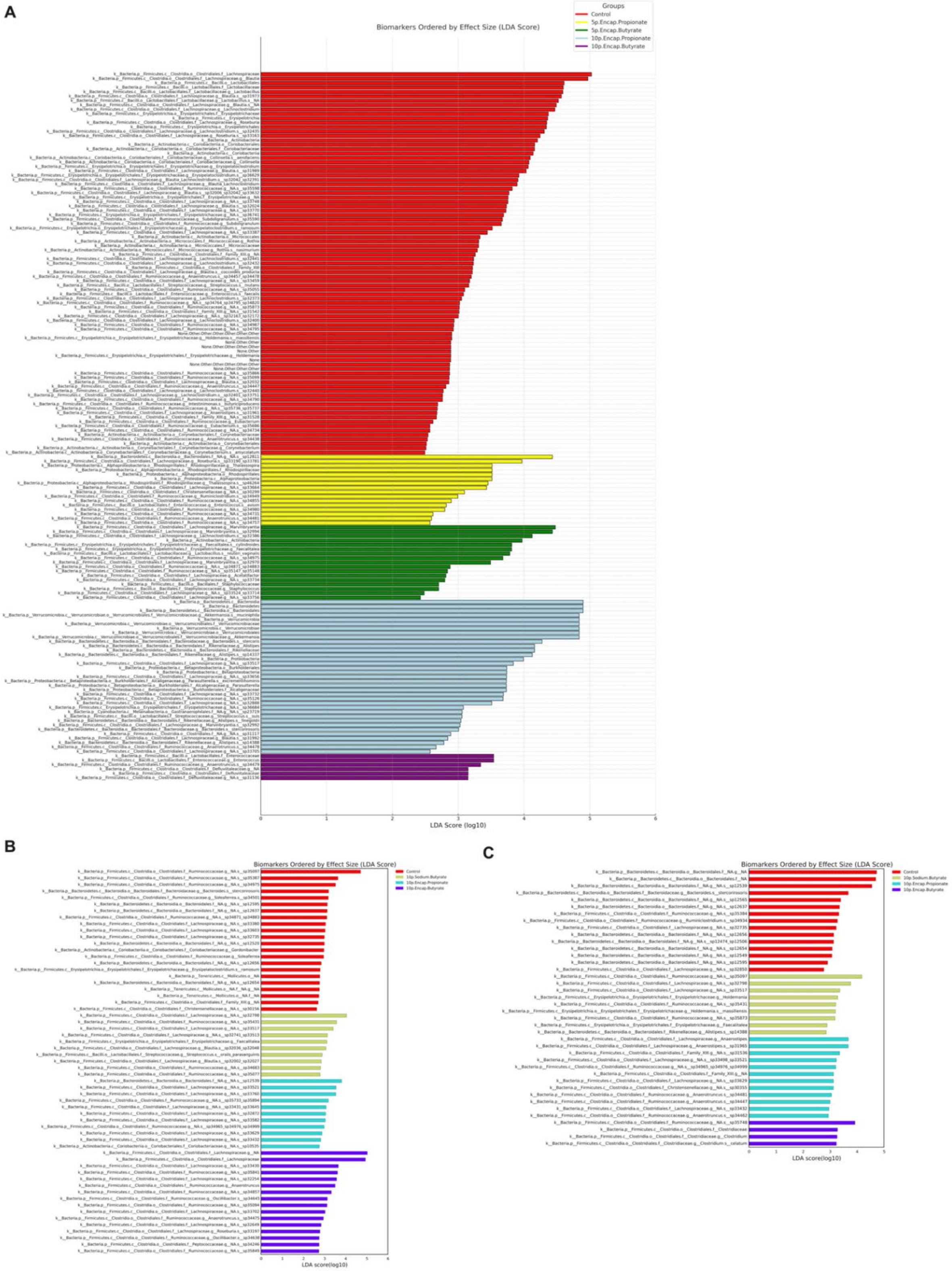

Figure S4.

V3V4_341f_p1_n6	TCGTCGGCAGCGTCAGATGTGTATAAGAGACAGNCCTACGGGDGGCWGCAG
V3V4_341f_p2_n4	TCGTCGGCAGCGTCAGATGTGTATAAGAGACAGNCCTAYGGGGCGCWGCAG
V3V4_341f_p3_n1	TCGTCGGCAGCGTCAGATGTGTATAAGAGACAGNCCTACGGGGTGCAGCAG
V3V4_341f_p4_n1	TCGTCGGCAGCGTCAGATGTGTATAAGAGACAGGCCTACGGGAGGCTGCAG
V3V4_806r_p1_n24	GTCTCGTGGGCTCGGAGATGTGTATAAGAGACAGNGACTACNVGGGTMTCTAATCC
V3V4_806r_p2_n4	GTCTCGTGGGCTCGGAGATGTGTATAAGAGACAGNGACTACNAGGGTATCTAATCC
V3V4_806r_p3_n3	GTCTCGTGGGCTCGGAGATGTGTATAAGAGACAGNGACTACDCAGGTCTCTAATCT
V3V4_806r_p4_n2	GTCTCGTGGGCTCGGAGATGTGTATAAGAGACAGNGAMTACCGGGGTTTCTAATCC
V3V4_806r_p5_n1	GTCTCGTGGGCTCGGAGATGTGTATAAGAGACAGNGACTACCAGGGTATCTAAGCC
